# Coating of ZnO Nanoparticle on Cotton Fabric to Create a Functional Textile with Enhanced Mechanical Properties

**DOI:** 10.3390/polym13162701

**Published:** 2021-08-13

**Authors:** Imana Shahrin Tania, Mohammad Ali

**Affiliations:** 1 Department of Mechanical Engineering, Bangladesh University of Engineering & Technology (BUET), Dhaka 1000, Bangladesh; 2 Department of Wet Process Engineering, Bangladesh University of Textiles (BUTEX), Dhaka 1208, Bangladesh

**Keywords:** ZnO nanoparticles, binder, mechanical properties, polyethylene wax emulsion, cotton fabric

## Abstract

The goal of this research is to develop a functional textile with better mechanical properties. Therefore, nano ZnO is synthesized, characterized, and applied to cotton fabric by mechanical thermo-fixation techniques. The synthesized nanoparticles are characterized by SEM and XRD analysis. The ZnO nanoparticle alone, ZnO nanoparticle with a binder, and ZnO with a binder and wax emulsion are then applied on cotton woven fabrics using three different recipes. The surface morphology of the treated fabric is studied using SEM and EDS. Antimicrobial activity, UV protection property, and crease resistance are all tested for their functional characteristics. In addition, all vital mechanical characteristics are assessed. The results suggest that using only nano ZnO or nano ZnO with a binder enhances functional features while deteriorating mechanical capabilities. Nano ZnO treatment with the third recipe, which includes the addition of an emulsion, on the other hand, significantly enhances mechanical and functional characteristics. Consequently, this study provides information to optimize the confidence of textile researchers and producers in using nano ZnO and understanding its features in key functional fabrics.

## 1. Introduction

Nanoscience and nanotechnology is a rapidly evolving interdisciplinary field that is being hailed as a new industrial revolution for the textile industry [1,2]. According to the national nanotechnology initiative (NNI), nanotechnology is characterized as using structures with at least one dimension of nanometer-scale for the construction of materials, devices, or systems with novel or significantly improved properties due to their nano-sizes [3,4]. The chemical and physical properties of a material change from the bulk to the nanometer scale. Therefore, a nanoparticle shows more reactivity and some other unique properties because of its high surface area to its volume ratio [5,6]. Nanotechnology is being more widely used as a result of its enormous economic potential [7]. The textile sector is also advancing by applying the numerous benefits of nanoparticles. Moreover, the consumer demand for long-lasting and sustainable practical clothing has opened the door for nanomaterials to be used in textiles [1]. The specific benefits of different nanoparticles are used in the textile field to manufacture advanced textiles in the functional and clothing sections. Several studies are being conducted to develop multifunctional textile materials that are coated with different nanoparticles. Nanoparticles such as silver (Ag), zinc oxide (ZnO), titanium dioxide (TiO_2_), and silicon dioxide (SiO_2_) have been used by researchers to impart functional property and meet the demand of modern markets [8,9,10]. For instance, a textile with high antibacterial activity was produced by utilizing the potentiality of silver nanoparticles [11,12,13]. More functional properties, such as ultraviolet protection, stain release, and antifungal activity, were achieved using TiO_2_ nanoparticles on textile fabric [14,15]. Other critical studies have been carried out to obtain multifunctional properties, such as self-cleaning, antimicrobial, antistatic, flame retardant, easy-care, UV defense, and water repellency [14,16,17,18,19].

However, ZnO nanoparticles have been found harmless and chemically stable during high temperatures exposing and photocatalytic oxidation [20,21]. Moreover, they have been highly attractive because of other remarkable potential applications in sunscreens, UV absorbers, anti-reflection coatings, photocatalysis, catalyst, solar cells, sensors, displays, piezoelectric devices, electro-acoustic, transducers, photodiodes, and UV light-emitting devices [8,22]. Moreover, they are a comparatively safe and inexpensive material that can be directly used for biomedical applications [23]. Due to these excellent properties of ZnO nanoparticles, much effort has been directed to the fabrication of ZnO with interesting morphologies and assemblies [24,25,26]. Furthermore, various methods are also used to apply ZnO nanostructures on textile materials, such as the hydrothermal route [27], ultrasonic irradiation technique, layer-by-layer deposition, pad-dry procedure, and sol–gel process [28,29,30].

Among many encouraging works related to the use of ZnO nanoparticles in the textile sector, there are also some limitations. Nano ZnO treatment on cotton fabric shows the degradation of its mechanical performance. Our recent paper revealed the degradation of tensile and tearing strength of woven fabric on both warp and weft directions owing to ZnO nano deposition [31]. Arputharaj [32] reported that ZnO nano-treated cotton fabric showed a 5.43% reduction in tensile strength for the warp direction. The nanoparticle type, treatment process, and fiber modification system are generally responsible for degrading important mechanical properties. Subash et al. [33] investigated the antimicrobial activity of ZnO nano-treated fabric, and they reported that the treated fabric showed excellent antibacterial activity against Gram-positive and Gram-negative bacteria.Nevertheless, this treatment lowered the essential mechanical characteristics, such as tensile strength, tearing strength, and abrasion resistance. Other related works of nano ZnO treatment on cotton fabric also reported similar reduction in the mechanical properties of textile fabric after using ZnO nanoparticles [4,34]. The wash durability of ZnO nano-treated fabric is also another challenging issue for nanoparticle researchers [35]. These issues may be the most significant impediment to using ZnO nanoparticles on cotton fabric. In this regard, chemical and auxiliaries employment may be emphasized to alleviate these challenges and increase nano deposition quality. As the mechanical property of the fabric deteriorates, it is necessary to use a chemical that provides smoothness, softness, and flexible handling qualities to the fabric. We know that fabric mobility [36] linked to the qualities listed is crucial in improving mechanical properties. The mentioned characteristics and a low friction coefficient ensure the uniform distribution of applied force across the whole fabric [37,38], resulting in improved mechanical performance [37,38]. Herein, a polyethylene wax emulsion is utilized to provide all outstanding smoothness and a soft handle to the fibers. From the supporting literature and technical data sheet [39], it is found that the wax emulsion, JinlubEco NP-825N (brand name), has extra lubrication and slippery effects and reduces the coefficient of friction. Further, it improves abrasion resistance and mechanical strength, thus increasing the weave and knitting productivity of yarns. This is the context for using polyethylene wax emulsion (JinlubEco NP-825N) combined with ZnO nanoparticles and a binder to increase fabric quality after nano ZnO deposition.

Additionally, the analysis of the essential physical properties of nano ZnO-treated fabric has not been well defined by the investigation of others. The mechanical properties are significant for cotton fabric related to the durability and serviceability of the textile. Furthermore, the improved mechanical properties play an essential role against various stresses and tension created on the fabric surface during coloration, finishing, cutting, sewing, and practical uses. Moreover, the comfortability and handling properties are also related to the stiffness of the fabric. Wrinkle recovery is regarded as a different functional quality and associated with the easy-care property of the fabric. The present study provides compiled and useable information regarding the above mechanical properties of nano ZnO-coated cotton fabric.

Zinc oxide nanoparticles are combined with auxiliary chemicals to obtain the optimal functional and mechanical qualities. A surfactant is also used in all recipes to reduce the surface tension of water and increase the wettability and hydrophilicity of the fabric. Using a binder and a wax emulsion for the immobilization of ZnO nanoparticles is a unique idea for improving the mechanical properties of cotton fabric. At the same time, it can create greater attention from textile manufacturers and eventually show a new dimension for quality fabric.

## 2. Experimental Section

### 2.1. Materials

The fabric of 100% cotton with a plain weave structure is collected from a local industry with 80 ends per inch (EPI) and 75 picks per inch (PPI), while the areal density is 168 g/m^2^. The raw cotton fabric is then scoured and bleached before nano-coating to impart absorbency and permanent whiteness. The chemical zinc sulfate heptahydrate (ZnSO_4_ • 7H_2_O, 99% purity) for synthesis is purchased from Merck Life Science Private Ltd., Mumbai, India. Ethanol and sodium hydroxide are purchased from Sigma Aldrich, Taufkirchen, Germany. Pretreatment chemicals: wetting agent, sequestering agent, detergent, caustic soda, and hydrogen peroxide are collected from Orient Chemical Ltd., Dhaka, Bangladesh. Polyethylene wax emulsion (brand name: Jinlub Eco NP-825N, character: softener, nonionic, yellowish liquid, water-soluble,) is collected from Jintex, Taiwan. A binder called OB-45 (nature: thermally cross-linkable, aqueous acrylate dispersion; appearance: low viscosity white milky liquid) is collected from Fortune top Pte Ltd. New Taipei City, Taiwan. Tubingen Chemicals, Comilla, Bangladesh, supplies a surfactant named Jingen DT HLF-18.

### 2.2. Synthesis of ZnO Nanoparticles

**Figure 1:**
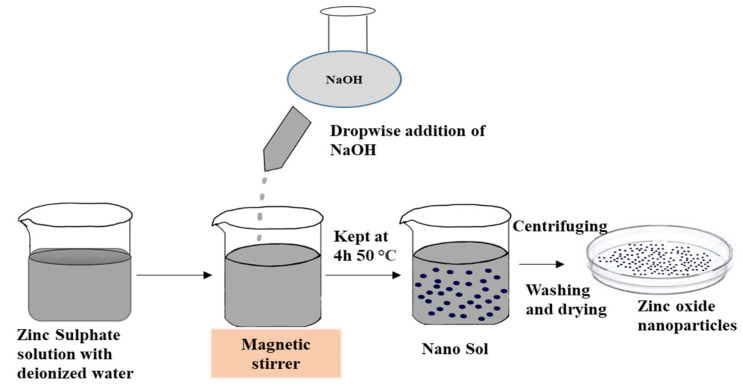
Experimental setup for the synthesis of ZnO nanoparticles.

The experimental setup of ZnO nanoparticle synthesis is shown in Figure 1. The wet chemical process is applied for the synthesis by following the method used by Kawano and Imai [40] with some modifications. An aqueous solution of 0.2 M zinc sulfate (ZnSO_4_) is made from zinc sulfate heptahydrate (ZnSO_4_ • 7H_2_O) in de-ionized water. A 25 mL of 0.2 M NaOH (pH = 3.8) solution is prepared separately with purified water. The reaction is then performed by slowly dropping NaOH into the ZnSO_4_ solution. The bath is placed on a magnetic stirrer for 30 min and kept for 4 h at 60 °C temperature. The nanoparticles are obtained by centrifuging and drying after washing.

### 2.3. Characterization of Nanoparticles

**Figure 2:**
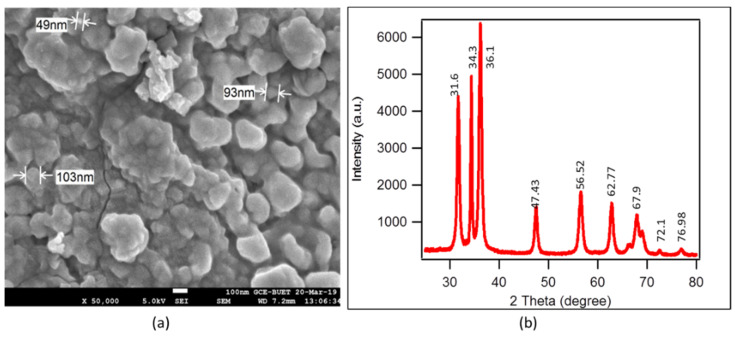
(**a**) SEM (scanning electron microscope) images of ZnO nanoparticles in ×50,000 magnification with size; (**b**) XRD (X-ray diffraction) pattern ZnO nanoparticles.

The synthesized nanoparticles are characterized by high-resolution scanning electron microscopy (SEM) and X-ray diffraction (XRD) analysis. A field emission electron microscope, JSM-6700F, Tokyo, Japan, determines the features, surface characteristics, and approximate size of the discrete nanoparticles. The sputtering device (Jeol JFC-1600 auto fine coater, Japan) creates a 75 nm thin platinum layer on our non-conductive cotton fabric in a vacuum environment before SEM analysis. Figure 2a shows the individual nanoparticle in various sizes and shapes in ×50,000 magnification. From this image, the size measuring software detects the approximate size of the nanoparticle. SEM analysis involves bombarding the surface of a sample with an intensely focused scanning electron beam generating a large number of secondary electrons whose strength is influenced by surface topography [11]. Particles of various shapes and diameters are visible in the SEM images. The X-ray diffractometer obtains the crystalline shape and size: Phillips, X’pert PRO, Holland. Figure 2b shows the XRD pattern of the ZnO nanoparticles. The measurement is carried out at a scanning rate of 8°/min in a 2θ range of 20–80°, using the Debye–Scherrer formula as Equation (1)

Particle Size = (0.9 × λ)/(d cosθ)(1)

where λ = 1.54060 Å(Angstrom), 0.9 × λ = 1.38654 Å, θ = 2θ/2, and d = the full width at the half maximum intensity of the peak.

This equation shows that the calculated mean size of the ZnO nanoparticle is 70 (±5) nm. The peaks of the XRD pattern are obtained at 2θ = 31.6 °, 34.3°, 36.1°, 47.43°, 56.52°, 62.77°, 67.9°, 72.1°, and 76.98° corresponding to the lattice planes (100), (002), (101), (102) (200), (112), (201) (004), and (202), respectively, which indicate the obvious formation of the ZnO nanoparticle. Our results strongly correlated with the standards (JCPDS) card file no-036–1451 for ZnO nanoparticles [41]. Similar peaks of ZnO nanoparticles are also found by the investigations of Sing et al. [42].

### 2.4. Application of ZnO Nanoparticles

**Figure 3:**
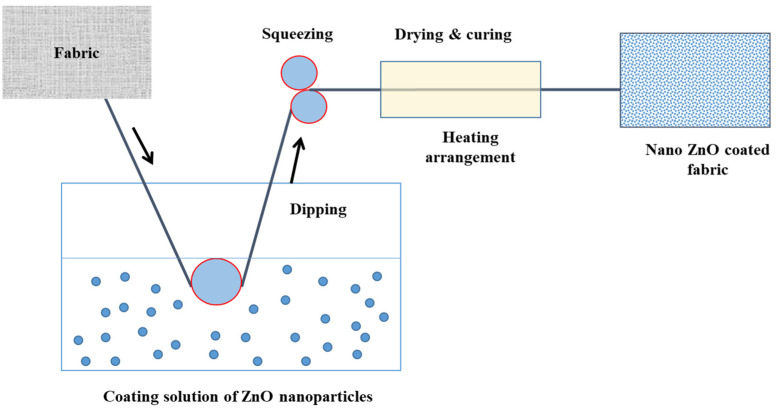
The sequence of thermo-fixation coating of nanoparticles on the fabric surface.

Mechanical thermo-fixation is used to adhere nanoparticles to cotton fabric (pad-dry-cure). Three distinct coating formulations containing the same quantity of nanoparticles and a surfactant are employed. Firstly, the fabric is coated with a 1% ZnO nanoparticle, denoted by the sample name NanoZnO-1. For the second coating, 1% ZnO nanoparticle solution and a binder are produced by stirring continuously for 10 min, and the resulting coated fabric is designated as a sample: NanoZnO-2. Finally, 1% ZnO nanoparticles, 10 g/L wax emulsion, and 1% binder are combined with propanol solution and stirred for 15 min at 60 °C. NanoZnO-3 is the sample name for the fabric coated with the third solution. Here, 73% pick-up is maintained throughout the padding, and the drying and curing conditions are 90 °C for 10 min and 150 °C for 5 min, respectively, resulting in the thermal fixation of ZnO nanoparticles into the fabric. The diagram of the thermo-fixation method [43] for nanoparticle application is shown in Figure 3.

### 2.5. Measurements and Analysis

The following measurements are carried out to analyze functional and mechanical properties: UV protection, antimicrobial activity, crease resistance, tensile strength, tearing strength, elongation, bending length, and frictional coefficient.

#### 2.5.1. Evaluation of Ultraviolet (UV) Protection

There are three categories of UV radiation of solar light: (i) UV-A of wavelength 320–400 nm, (ii) UV-B of 280–320 nm, and (iii) UV-C of 200–280 nm. The UV-A rays are the least harmful; the UV-B rays are the more powerful and harmful form of radiation, and they are the most common cause of sunburn, aging, wrinkling, and skin cancer. The UV-C rays are the shortest and the most harmful, but, fortunately, most of them are absorbed by the atmosphere’s ozone layer. Therefore, the concerning regions for the evaluation of sun protection arise on UV-A and UV-B regions [44]. Protection against the harmful effects of UV rays by using functional textile material is an appreciable way to prevent skin cancer or other related skin diseases caused by the UV rays of the Sun [45]. Textile fabric gives protection by absorbing and blocking the penetration of UV rays [46]. The UV protection of our treated and untreated fabric is measured by the PerkinElmer UV visible machine from the USA, model-LAMBDA 1050+. This instrument provides the transmittance curve of every sample. By analyzing the transmittance curve, the UV protection power is evaluated.

#### 2.5.2. Analysis of Antimicrobial Activity

The antimicrobial activity of nano-treated and untreated fabric is quantitatively evaluated by the standard test method [47]. The study is performed against Gram-positive *S. aureus* and Gram-negative *E. coli* by the colony counting method, and the results are expressed by a bacterial reduction percentage (R%). For this test, the number of viable species in suspension is estimated, and the percentage reduction is measured based on recoveries from the untreated sample. This approach is intended for a surfaces with 50–100% reduction capacity for the required contact time [48]. The following formula calculates the percent of bacteria killed within the designated time in Equation (2):

The bacterial reduction percentage

(2) $$(R\%)=\frac{\mathrm{No}-\mathrm{Nt}\;}{\mathrm{No}}\times 100$$

No = number of colonies in control and Nt = number of colonies after the selected hour of introducing samples. Here, the samples are tested both after 1 h and 24 h of contact time.

#### 2.5.3. Crease Resistance

A crease is a broken line, mark, or fold on a fabric caused by sharp folding. It is formed when the fabric is distorted so that part of it is stretched beyond its elastic recovery [49]. Crease resistance is a property of fabric that prevents fabric from creasing and is generally measured by the recovery angle of the fabric after creasing. A higher angle of recovery indicates more resistance to the crease. Thus, it is a quantitative measurement of the easy-care property of the fabric. In the current research, the crease recovery angle of untreated and treated fabric is measured using the AATCC standard test method [50].

#### 2.5.4. Mechanical Properties

The tensile strength is measured by the ISO 13934-1:2013 test method, and tearing strength is measured by ISO13937-2:2000. The Titan Universal Strength Tester of James Heal is used for the test. Measurements are performed in both the warp and weft directions of the fabrics. Average values are obtained from the measurements of three samples. Fabric stiffness expressed by bending length is determined by ASTM D1388 using the Shirley stiffness tester. The frictional property is measured by the Fabric Touch Tester of SDL Atlas, USA. All experimental samples are conditioned in the standard atmosphere at 20 ± 5 °C and 65 ± 2% relative humidity for 12 h in a conditioning laboratory before every test.

## 3. Results and Discussion

### 3.1. Assessment of Ultraviolet (UV) Protection

**Figure 4:**
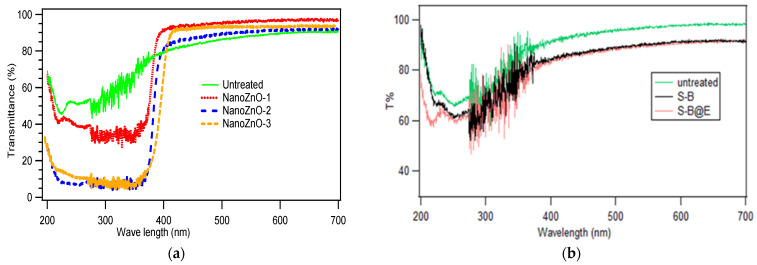
UV transmittance curve of (**a**) untreated fabric and different ZnO nano-coated fabric; (**b**) untreated fabric, only binder-treated (S-B) fabric, and binder with emulsifier-treated fabric (S-B@E).

Ultraviolet protection is assessed by the UV transmittance curve of untreated and treated fabric measured using a UV visible spectrophotometer by selecting a wavelength region of 200–700 nm. The obtained results are presented in Figure 4a. The curve clearly shows the amount of transmittance in percentage by treated and untreated fabrics. The approximate quantitative value of transmittance is listed in Table 1 based on the transmittance curve. The typical UPF (ultraviolet protection factor) ranges are also presented in Table 1, which are determined by following the literature [51] and the standard AS/NZS 4399. It is found that the untreated fabric transmitted about 60% of UV light at a wavelength of ~300 nm, which is the most harmful to the skin.

On the other hand, the sample NanoZnO-1, which contains 1% ZnO nanoparticles, exhibits roughly 35% UV transmittance in that region. This indicates that the ZnO nanoparticle enables the fabric to block 65% of UV rays. The coated fabric NanoZnO-2 shows nearly ~6% transmittance of the UV spectrum. Hence, it blocks 94% of incident light on the UV-B region. The transmittance nature on UV-A of 200–280 nm is similar, as it blocks ~92% of incident light. Therefore, the binder fixes more ZnO nanoparticles on cotton fabric, which brings a higher UV blockage. Eventually, the UV protection ability of treated fabric with the third recipe indicates the highest UV blockage at 280–320 nm. NanoZno-3 provides protection of around 95% UV, as it transmits only ~5% UV rays. This transmittance is similar to the UPF range 25+ designated to the very good UV protection category [52,53]. According to the literature [41], 30+ UPF refers to very good UV protection with 6% UV transmission. However, our treated fabric NanoZnO-3 provides approximately 30 ± 3 (average UPF ranges), indicating that we discovered outstanding UV protection. When all of the data in Table 1 are compared, it is clear that the ZnO nano-coating can increase the UV protection of cotton fabric.

Significant improvements have been made by using a binder as a fixing agent for the deposition of nanoparticles. In addition, ZnO has a well-defined sun protection power [45,54] and is capable of reflecting both UV-A (320–400 nm) and UV-B (280–320 nm) wavelengths of sunlight [55]. As a result, the coated fabric in this study, which is made up of nano ZnO particles measuring 70 nm (about), exhibits strong resistance to UV light blocking. However, our findings are consistent with the previous investigation of nano ZnO on fabric. Yuwanda and Punnama [56] used poly 4-styrenesulfonic acid to immobilize ZnO nanoparticles on cotton fabrics and obtained similar UV protection. Likewise, AS/NZS 4399 defined three levels of protection: excellent, very good, and good [57], which are also listed in Table 1 for our working samples. Finally, it should be noted that our result of significantly improved UV protection of cotton fabric is the attribute of the shielding effect of ZnO nanoparticles [56]. However, the two auxiliary chemical binders and wax emulsion used in this study have a minor impact on UV protection, as it is found from Figure 4b that the samples with the binder (S-B) only and the binder with an emulsifier (S-B@E) demonstrate minimal changes in UV transmittance (T%) than that of the untreated fabric. The binder only increases the nano deposition; therefore, the samples NanoZnO-2 and NanoZnO-3 show higher protection.

### 3.2. Assessment of Antimicrobial Activity

**Figure 5:**
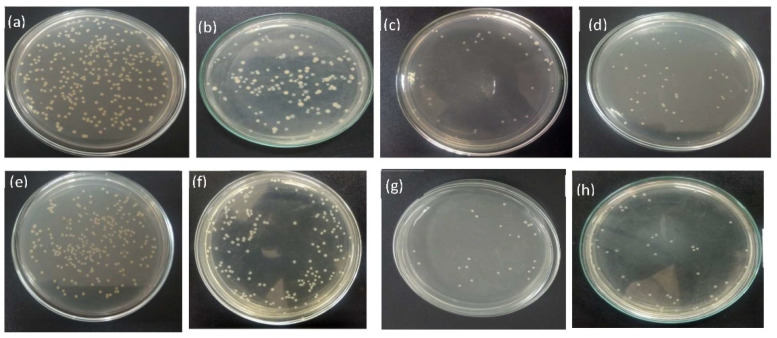
Bacterial reduction disk images of 1 h contact time for (**a**) untreated fabric, (**b**) NanoZnO-1, (**c**) NanoZnO-2, and (**d**) NanoZnO-3 for *S. aureus*; (**e**) untreated fabric, (**f**) NanoZnO-1, (**g**) NanoZnO-2, and (**h**) NanoZnO-3 for *E.coli*.

**Figure 6:**
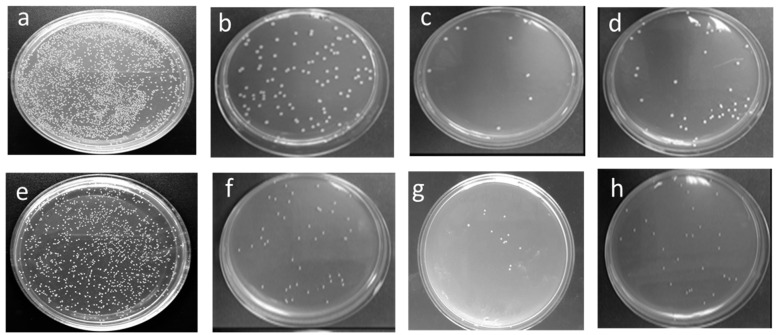
Bacterial reduction disk images after 24 h contact time for (**a**) untreated fabric, (**b**) NanoZnO-1, (**c**) NanoZnO-2, and (**d**) NanoZnO-3 for *S. aureus* and (**e**) untreated fabric, (**f**) NanoZnO-1, (**g**) NanoZnO-2, and (**h**) NanoZnO-3 for *E.coli*.

The killing of two bacteria for the action of nano ZnO-coated fabric by three different recipes is presented as the percentage of bacterial reduction (R%). The obtained results of R% are shown in Table 2. The agar plate, having a bacterial reduction image of all treated and untreated fabrics, is shown in Figure 5 and Figure 6. The results show that all three coated fabrics give a notable amount of bacterial reduction. The coated sample NanoZnO-1 achieves 58.76% reduction for *S. aureus* and 50.54% for *E. coli* after 1 h of contact time. The sample NanoZnO-2 reduces the highest amount of bacteria with 90.43% reduction for *S. aureus* and 86.14% for *E.coli*. The third sample, NanoZnO-3, also shows a bacterial reduction of 84.47% for *S. aureus* and 80.2% for *E.coli* in unwashed conditions after 1 h of contact killing. The reduction increases significantly after 24 h of the contact period. The treated fabric NanoZnO-2 and NonoZnO-3 show ~99% reduction in both bacteria after 24 h.

Figures 8 and 9 show the higher amount of nano zinc particles in sample NonoZnO-2. Therefore, the increasing amount of nanoparticles causes the higher antibacterial activity of the second sample of NanoZnO-2. Anita et al. [33] found 99% reduction (R%) for *S. aureus* and 80% for *E. coli* for the ZnO (2%) nano-treated cotton fabric. Due to the use of a binder in our study, we achieve ~99% bacterial reduction for both *S. aureus* and *E.coli* bacteria, even when using 1% nano ZnO. The contribution of the binder on antimicrobial activity is also proved by our previous work [31]. El-Nahhal et al. [58] found around 90–93% bacterial reduction on cotton fabric for ZnO nanoparticles and identified low wash durability of antimicrobial activity. Compared with the results of other related studies, our results of bacterial reduction are up to the required standard.

Nevertheless, due to the use of a binder, our wash stability is higher, as shown in Table 2. Furthermore, the literature supports that the acrylic binder fixes more nanoparticles, resulting in intensive antibacterial action [59]. However, from the second and third treated samples, it can be found that the binder has a significant contribution to the deposition of ZnO nanoparticles on the surface of the textile fabric. Eventually, these samples develop outstanding properties of antimicrobial activity. The efficient antimicrobial activity of ZnO nanoparticles is explained as the generation of reactive surface oxygen, which kills the bacteria by damaging the cell membrane of microbes [60]. Therefore, utilizing the present technique for the deposition of sufficient ZnO nanoparticles is an interesting way to participate in the excellent antimicrobial activity.

### 3.3. Effect of ZnO Nanoparticles on Crease Resistance

**Figure 7:**
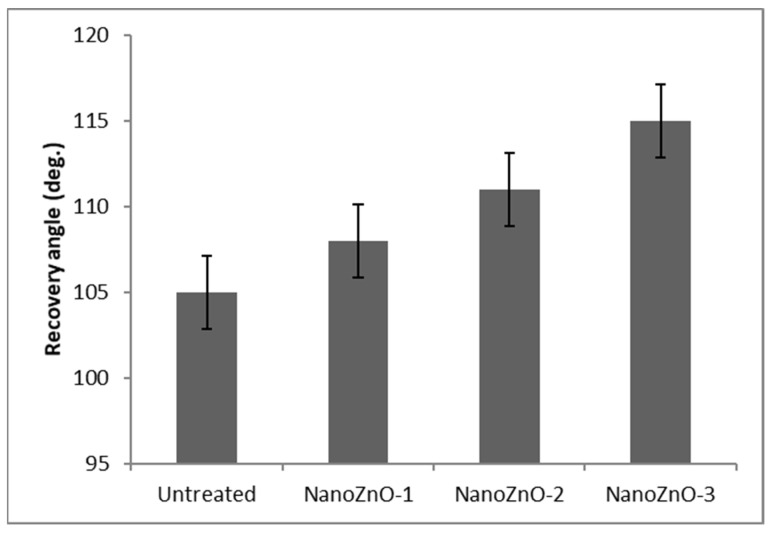
Crease recovery of untreated and ZnO nano-treated fabric.

The recovery angle of the different samples measured in degrees is shown in Figure 7, showing that the ZnO nanoparticle has some appreciable effects on the crease recovery angle of cotton fabric. The untreated fabric has 105° (degree) of recovery angle and NanoZnO-1 has 108°. Hence, the first coating with 1% ZnO nanoparticle shows a 3% improvement in recovery after creasing. The second sample, NanoZnO-2, shows a 5.7% improvement in crease recovery. Finally, the highest amount of crease recovery is obtained by NanoZnO-3, which delivers around 10% improvement in crease recovery compared with that of the untreated fabric. Thus, crease recovery and the easy-care property can be improved by the nano ZnO coating and the binder and emulsion. It is known that cotton fabric is inelastic and contains so many free hydroxyl (-OH) groups in its structure that are responsible for easy crease formation [61,62]. For the coated fabric of this research, ZnO nanoparticles easily penetrate the fabric pores. They are affixed tightly on the surface by the binder, and, consequently, it is not easily wrinkled or creased after treatment. Moreover, the emulsion penetrates the cellulose polymer, blocks the free hydroxyl group, and improves fiber mobility. Thus, the nano ZnO-treated fabric shows better performance than the untreated fabric, NanoZnO-2 performs much better, and NanoZnO-3 performs the best on crease recovery among the samples.

### 3.4. Evaluation of Mechanical Properties

The obtained results of tensile strength, elongation, tearing strength, bending length, and friction coefficient are shown in Table 3. From the table, it is found that the two samples NanoZnO-1 and NanoZnO-2 show a significant reduction in tensile strength compared with that of the untreated fabric. The reduction in tensile strength is more for NanoZnO-2, where the binder is used as a fixing agent. It can be observed that NanoZnO-1 treated with only ZnO nanoparticles reduces ~5.6% strength in the warp direction and ~3.9% in the weft direction. The use of the binder increases ZnO deposition, decreasing ~10.5% of tensile strength in the warp direction and ~12.23% in the weft direction.

On the other hand, for the sample of NanoZnO-3, the tensile strength rises to ~795.20 N compared to ~730.52 N of the untreated fabric in the warp direction and ~616.32 N compared to ~572.6 N of the untreated fabric in the weft direction, which are 8.8% and 7.6% higher in the warp and weft directions, respectively, than that of the untreated fabric. In contrast, Anita et al. [33] and Yadav et al. [4] reported that nano ZnO deposition reduced the tensile strength of the cotton fabric, where the authors did not use any other auxiliaries for the deposition of nano ZnO. Herein, polyethylene wax emulsion is used along with a binder and 1% ZnO nanoparticle. Therefore, the emulsion has a significant effect on the polymeric bonding of fiber molecules and improves the tensile strength. However, from the analysis of the tensile strength test of the four samples, it is observed that the ZnO nanoparticles deposited on the cotton fabric decrease the tensile strength in both warp and weft directions. The higher deposition of the ZnO nanoparticle causes more reduction in tensile strength as per the results of samples NanoZnO-1 and NanoZnO-2. For resolving the degradation of tensile strength, one innovative idea is the use of polyethylene wax emulsion during nano treatment similarly to NanoZnO-3, which can be attributed to an 8.8% increase in tensile strength in the warp direction and 7.6% in the weft direction compared to that of the untreated fabric as shown in Table 3. Similar trends of tensile strength can be found for elongation and tearing strength, as shown in Table 3. The nano ZnO coating without emulsion reduces these two properties for samples NanoZnO-1 and NanoZnO-2. Quantitatively, NanoZnO-1 has a reduction of 4.3%, and NanoZnO-2 has a decrease of 12.3% in elongation along the warp direction compared than that of the untreated fabric. The tearing strength is also reduced in both warp and weft directions for samples NanoZnO-1 and NanoZnO-2. On the other hand, the sample NanoZnO-3 improves the elongation and tearing strength in both warp and weft directions, which are, no doubt, very noticeable results.

For textile fabric, bending length is a measure of stiffness, i.e., if the bending length is higher, the fabric stiffness is higher. Therefore, the nano ZnO treatment slightly increases the stiffness of the cotton fabric, as can be observed in Table 3. Again, the nano deposition with the binder makes the fabric stiffer, as NanoZnO-2 achieves a bending length of 2.1 cm, and that is 16% higher than that of the untreated fabric. However, the sample NanoZnO-3 shows a 22.2% reduction in bending length than that of the untreated fabric due to its softness, smoothness, and mobility imparted by the polyethylene wax emulsion. Therefore, increasing the bending length for the first and second nano-treated samples might marginally make the fabric stiffer, thereby reducing fabric mobility. Consequently, the force imparted could not be readily distributed across the whole fabric, which might be the reason for the reduction in tensile strength. The reduction in tensile strength was also confirmed by the literature [63] through the overall chemical finishing of the textile fabric.

From the results of frictional resistance in Table 3, the ZnO nanoparticle decreases the frictional co-efficient of all the nano-treated samples compared with the untreated fabric. An excellent feature is obtained for the sample NanoZnO-3, where the use of emulsion reduces both static and kinetic frictional resistance remarkably. This type of nano-coating will help textile fabric resist mechanical damage during the wet process and garment preparation. Most importantly, due to the reduction in kinetic friction, the cutting speed can be increased, and the low static friction improves the sewing production rate.

### 3.5. Analysis of Surface Morphology

**Figure 8:**
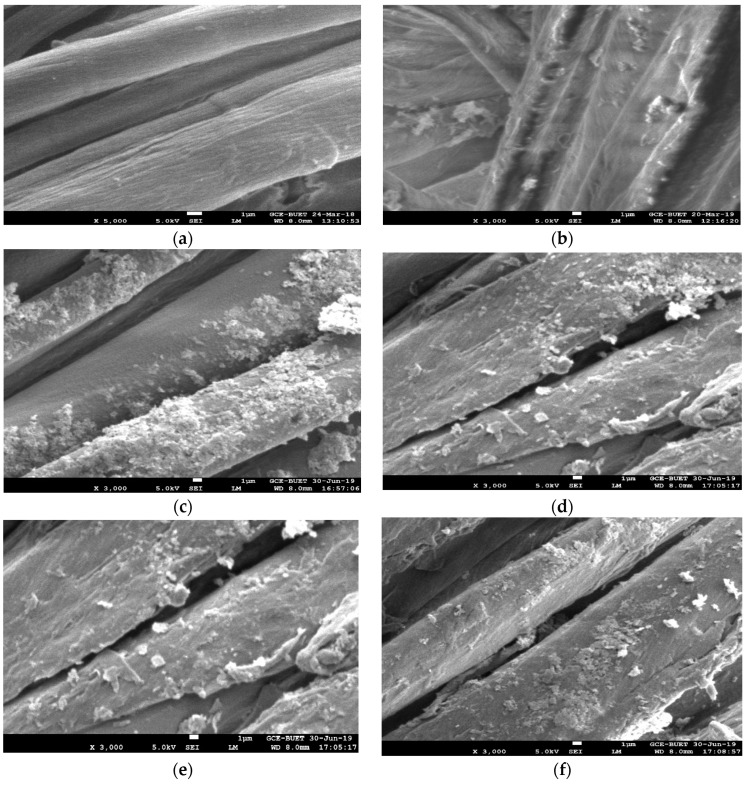
SEM images of different samples: (**a**) untreated fabric, (**b**) NanoZnO-1, (**c**) NanoZnO-2, (**d**) NanoZnO-3, (**e**) NanoZnO-2 after washing, and (**f**) NanoZnO-3 after washing.

**Figure 9:**
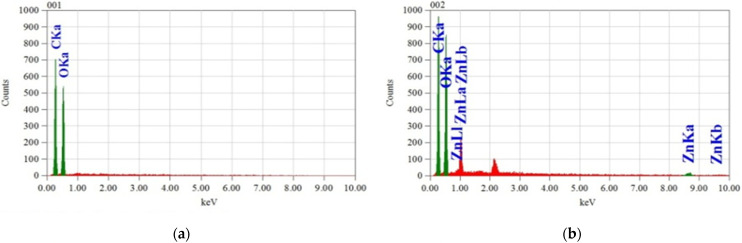

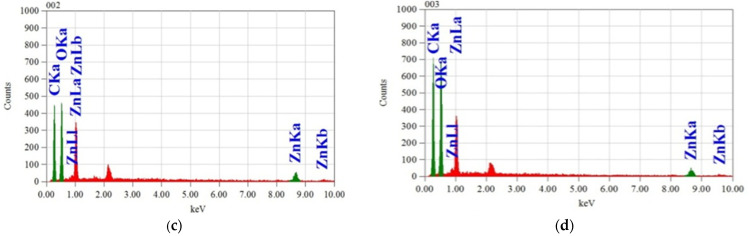
EDS of different samples: (**a**) untreated fabric, (**b**) NanoZnO-1, (**c**) NanoZnO-2, and (**d**) NanoZnO-3.

The morphological change in the untreated and nano ZnO-treated fabric is observed by SEM, as shown in Figure 8a–f, to support the analyses of the results obtained by the present investigation. The samples are enlarged with ×3000 magnification for the betterment of observation. The untreated fabric shows a smooth and clear image of the cotton fiber structure in Figure 8a, whereas the treated fabric shows a sufficient amount of nanoparticles on the fabric surface in Figure 8b–d. Additionally, Figure 8e; Figure 8f represents the SEM images of NanoZnO-2 and NanoZnO-3 after five washes, followed by the standard test method [64]. The image shows a sufficient amount of ZnO nanoparticles on the surface of the washed fabric that happens due to a binder. The binder improves bonding and imparts longevity of nano deposition. The EDS (energy dispersive spectroscopy) images are depicted in Figure 9a–d. Both SEM and EDS analyses indicate that the nano-treated sample with only nano ZnO contains fewer nanoparticles than the other treated fabrics. The SEM analysis supports all of the obtained results, i.e., the higher concentration of nano ZnO causes an improvement in functional activity but degrades mechanical properties. However, the use of polyethylene wax emulsion and the binder on the nano ZnO-coated fabrics ensures a sufficient amount of nanoparticle deposition to improve functional and mechanical properties.

## 4. Conclusions

Cotton fabric is coated by synthesized ZnO nanoparticles following the mechanical thermo-fixation method. Three different coating recipes are used with the aim of improving the mechanical properties as well as functional qualities. The nano deposition on the fabric surface is proved by the SEM and EDS images. Though the ZnO nanoparticle is used to develop antimicrobial activity in making medical cloth, the degradation of mechanical properties is the drawback of nano ZnO treatment. For the improvement of those mechanical properties, polyethylene wax emulsion is added with nano ZnO. It is an agent that forms a flexible film on the fabric surface, makes the fabric soft with a smooth coating, and improves mechanical properties. Excellent and encouraging results can be found for the antibacterial activity, where the nano treatment with the binder shows an improvement of ~99% bacterial reduction for *S. aureus* and ~97% for *E.coli* after 24 h. The nano ZnO with the binder and emulsion shows ~98% bacterial reduction for *S. aureus* and ~97% for *E.coli* with outstanding wash durability. The UV protection is also excellent for nano ZnO with the binder and nano ZnO with the binder and emulsion, restricting approximately 90% of UV rays of the 250–375 nm wavelength region. It is found that the use of the binder for the coating of nano ZnO degrades tensile strength, elongation, tearing strength, and the frictional coefficient.

In contrast, excellent improvement of these properties is found when polyethylene wax emulsion and the binder are used to coat nano ZnO. Thus, this study demonstrates the improvement of the functional properties of cotton fabric as well as the mechanical properties. It promotes longevity, serviceability, comfortability, and high-quality finished cotton fabric. Moreover, the UV protective textile produced by coating with nano ZnO, binder, and emulsion becomes an essential, valuable item as curtains, awnings, and summer clothes along with its application in the medical sector.

## Figures and Tables

**Table 1:** Average UV transmittance (%) of different wavelengths with UPF range.

Sample	Transmittance (%)			Measured UPF with Respect to Average Transmittance%	UV Protection Category *
	UV-A (320–400) nm	UV-B (280–320) nm	UV-C (200–280) nm		
Untreated	70	60	52	5 ± 2	Poor
NanoZnO-1	42	35	34	8 ± 3	Moderate
NanoZnO-2	7	6	8	28 ± 4	Very good
NanoZnO-3	6	5	10	30 ± 3	Very good

* Category of UV protection assessed as per AS/NZS 4399: 1996 [57].

**Table 2:** Bacterial reduction on untreated and nano-treated fabric for *S. aureus* and *E.coli*.

Sample	Bacterial Reduction R% after 1 h Contact Time		Bacterial Reduction R% after the 24 h Contact Time	
	*S. aureus*	*E. coli*	*S. aureus*	*E. coli*
Untreated	--	--	--	--
NanoZnO-1				
Unwashed	58.76	50.54	68.30	57.11
5 wash	40.22	30.11	52.40	40.40
10 wash	25.46	18.23	31.14	23.6
NanoZnO-2				
Unwashed	90.43	86.14	99.50	97.10
5 wash	86.24	83.60	98.16	96.01
10 wash	85.46	83.00	96.20	93.34
NanoZnO-3				
Unwashed	84.47	80.20	98.12	97.45
5 wash	81.53	77.61	96.15	95.01
10 wash	82.10	76.48	94.4	92.34

**Table 3:** Different mechanical properties of untreated and nano ZnO-treated cotton fabric with mean deviation.

Sample	Tensile Strength Breaking Force (N)		Elongation (%)		Tearing Strength (N)		Bending Length (cm)	Frictional Coefficient	
	Warp	Weft	Warp	Weft	Warp	Weft		Static	Kinetic
Untreated	730.52 ± 9	572.60 ± 6	21.10 ± 3	14.75 ± 3	9.11 ± 2	4.3 ± 1	1.8 ± 1	0.35	0.32
NanoZnO-1	689.3 ± 8	550.20 ± 5	20.2 ± 4	15.10 ± 2	8.30 ± 3	3.6 ± 1	1.9 ± 1	0.31	0.28
NanoZnO-2	654.15 ± 7	502.54 ± 5	18.5 ± 3	13.22 ± 3	6.23 ± 3	3.1 ± 2	2.1 ± 1	0.30	0.27
NanoZnO-3	795.20 ± 9	616.32 ± 7	22.5 ± 5	18.63 ± 4	12.45 ± 4	8.6 ± 4	1.4 ± 1	0.23	0.20

## Data Availability

Not applicable.

## Abbreviations

SEM	Scanning Electron Microscope
EDS	Energy Dispersive Spectroscopy
XRD	X-Ray Dispersive Spectroscopy
UPF	Ultraviolet Protection Factor
UV	Ultraviolet Ray
T%	Transmission Percentage
AATCC	The American Association of Textile Chemists and Colorists
